# Clustering of Brain Function Network Based on Attribute and Structural Information and Its Application in Brain Diseases

**DOI:** 10.3389/fninf.2019.00079

**Published:** 2020-02-07

**Authors:** Xiaohong Cui, Jihai Xiao, Hao Guo, Bin Wang, Dandan Li, Yan Niu, Jie Xiang, Junjie Chen

**Affiliations:** College of Information and Computer, Taiyuan University of Technology, Taiyuan, China

**Keywords:** graph mining, similarity, sub-network kernels, spectral clustering, Alzheimer's disease

## Abstract

At present, the diagnosis of brain disease is mainly based on the self-reported symptoms and clinical signs of the patient, which can easily lead to psychiatrists' bias. The purpose of this study is to develop a brain network clustering model to accurately identify brain diseases based on resting state functional magnetic resonance imaging (fMRI) in the absence of clinical information. We use cosine similarity and sub-network kernels to measure attribute similarity and structure similarity, respectively. By integrating the structure similarity and attribute similarity into one matrix, spectral clustering is used to achieve brain network clustering. Finally, we evaluate this method on three diseases: Alzheimer's disease, Bipolar disorder patients, and Schizophrenia. The performance of methods is evaluated by measuring clustering consistency. Clustering consistency is similar to clustering accuracy, which is used to evaluate the consistency between the clustering labels and clinical diagnostic labels of the subjects. The experimental results show that our proposed method can significantly improve clustering performance, with a consistency of 60.6% for Alzheimer's disease, with a consistency of 100% for Schizophrenia, with a consistency of 100% for Bipolar disorder patients.

## Introduction

In recent years, graph mining has become a popular research field and has been widely used in computer networks (Zou et al., 2017), social network analysis (Halder et al., 2016) and computational biology (Zhang et al., 2017). In addition, many new kinds of data can be represented as graphs, such as functional magnetic resonance imaging (fMRI) data. Using fMRI data we can construct the brain functional connectivity network in which each node represents a brain region and each edge represents the functional connectivity between two brain regions (Kong and Yu, 2014). These brain networks provide us with a means to explore the function of the human brain and provide valuable information for clinical diagnosis of neurological diseases, such as Alzheimer's disease (AD), Bipolar disorder patients (BD), and Schizophrenia (SC). Therefore, brain network analysis based on graph mining has become a new research hotspot and attracted increasingly more researchers.

In brain science studies, some brain network of subjects were given, some of whom suffered from certain brain diseases (such as AD or BD), while the other group was a normal control group without any brain disease. The next task is to distinguish the two types of subjects accurately. In this problem, most of the researchers are based on the assumption that brain networks with similar structures have similar functional characteristics. Therefore, the key problem is how to measure the similarity of brain network.

The existing similarity measure of brain networks can be classified into two main classes (Mheich et al., 2019): (1) the statistical comparison, where various graph theoretical metrics (such as efficiency and betweenness) can also be estimated at node or edge level of the compared networks (Bullmore and Bassett, 2011). These metrics are then quantitatively compared between two groups of networks via statistical tests. (2) Graph matching, where the main purpose is to quantify a similarity score between two brain networks by considering structure distance. This method includes: edit distances, hamming distance (Gao et al., 2010) and kernel methods (Shervashidze et al., 2011).

In this paper, by combining the above two class methods, a similarity measurement method of brain network based on node attribute similarity and structural similarity is proposed, and the method is applied to the clustering of brain network. We use cosine similarity and sub-network kernels to measure attribute similarity and structure similarity, respectively. By integrating the structure similarity and attribute similarity into one matrix, spectral clustering is used to achieve brain network clustering.

**Figure 1:**
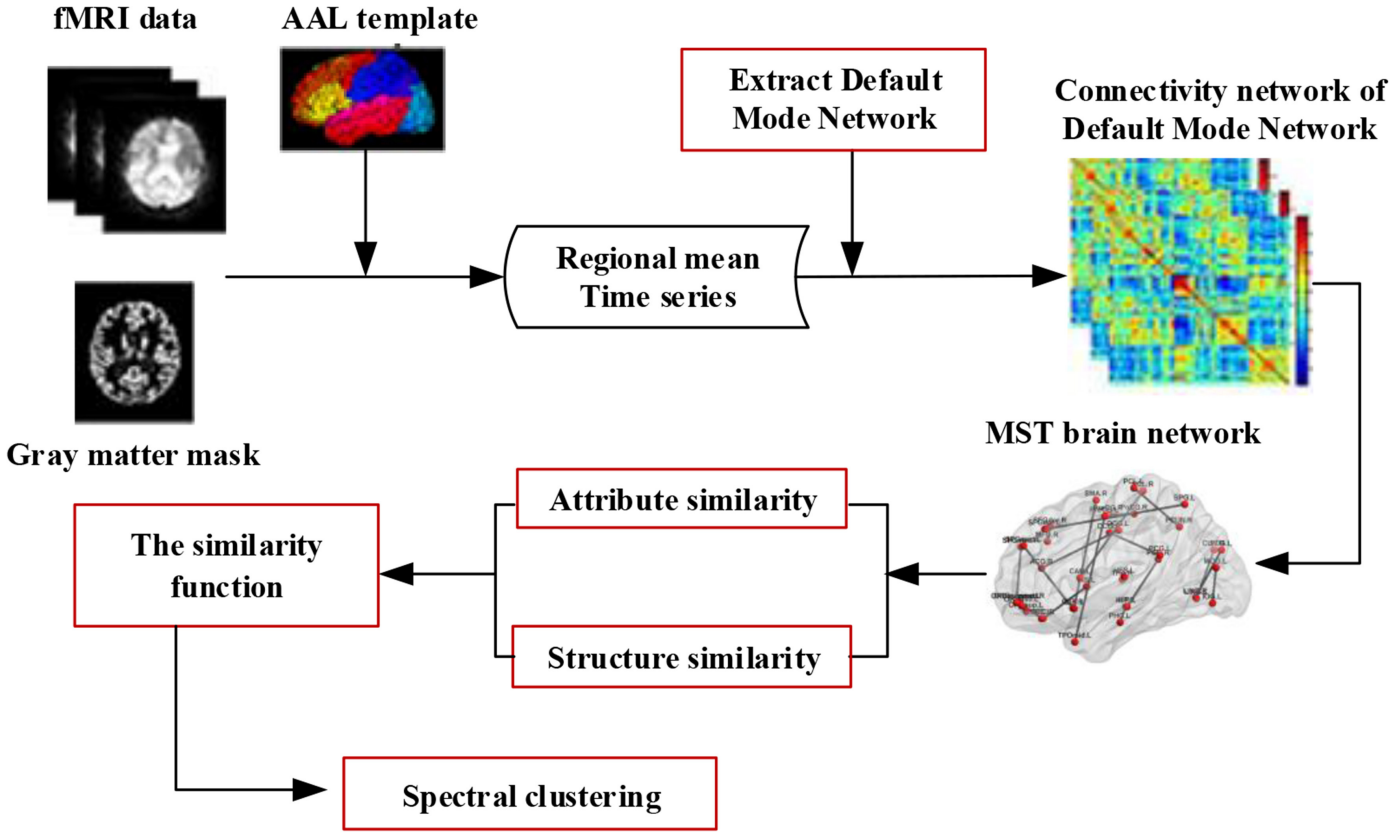
The framework of spectral clustering based on brain network. Firstly, the fMRI data is preprocessed, and the minimum spanning tree (MST) network of the default mode network (DMN) is constructed. Then, two different types of similarity (attribute similarity and structural similarity) are calculated. Finally, the two kinds of similarity are effectively combined and the brain network clustering is carried out.

This framework is illustrated in Figure 1. Specifically, for each brain connectivity network, we first preprocess the fMRI data and construct a minimum spanning tree (MST) network of the Default Mode Network (DMN), then compute two different types of similarity (attribute similarity and structure similarity) and effectively integrate these for spectral clustering. Finally, we evaluate the proposed method on three datasets. One dataset was from Alzheimer's Disease Neuroimaging Initiative (ADNI) dataset. The other two dataset were selected from the UCLA Consortium for Neuropsychiatric Phenomics LA5c Study, and the study was approved by the UCLA Institutional Review Board. Cluster consistency is used to evaluate the performance of the method. The cluster consistency is similar to the clustering accuracy, which reflects the consistency between the cluster results and the clinical diagnosis results. It can be seen from the experimental results that the consistency of the proposed brain network clustering algorithm is high, which shows that the clustering of the brain network can be accurately realized without the clinical diagnosis information.

## Materials and Methods

### Data Acquisition and Preprocessing

The data used in this study was from three datasets. One was the Alzheimer's Disease Neuroimaging Initiative (ADNI) database (http://adni.loni.usc.edu/). The other two were obtained from a public database, openfMRI dataset (https://www.openfmri.org/). Its accession number is ds000030.

In the ADNI database, 109 subjects (48 AD patients and 61 NC) were selected for analysis. Of these, 55 participants (26 AD patients and 29 NCS) were selected from ADNI-2. These data meet the following parameter settings: repetition time (TR) = 3,000 ms; echo time (TE) = 30 ms; slice thickness = 3.3 mm; flip angle = 80°; slice number = 48 and 140 time points. During scanning, all the subjects were instructed to keep their eyes closed. Another 54 participants (22 AD patients and 32 NCs) were selected from ADNI-3. These data meet the following parameter settings: repetition time (TR) = 3,000 ms; echo time (TE) = 30 ms; slice thickness = 3.4 mm; flip angle = 90°; slice number = 48 and 197 time points. Table 1 shows the demographic information of the participants.

**Table 1:** Demographic information of study participants.

**Group**	**AD**	**NC**
No. of subjects (M/F)	23/25	24/37
Age (mean ± SD)	74.0± 8.7	73.0± 7.3
MMSE (mean ± SD)	22.8 ± 2.5	28.9 ± 1.2
CDR (mean ± SD)	0.8 ± 0.2	0.0 ± 0.0

*AD, Alzheimer's Disease patients; NC, Normal Control; MMSE, Mini-Mental State Examination; CDR, Clinical Dementia Rating; M, Male; F, Female*.

In openfMRI database, 49 bipolar disorder patients (BD), 50 schizophrenia (SC) and 49 age- and gender-matched normal subjects (NC) were selected for analyzing. Data meets the following parameter settings: repetition time (TR) = 2,000 ms; echo time (TE) = 30 ms; slice thickness = 4 mm; flip angle = 90°; slice number = 34 and 152 time points. The detailed demographics and clinical features of the patients and normal controls are described in Table 2.

**Table 2:** Demographic information of study participants.

**Group**	**BD**	**SC**	**NC**
No. of subjects (M/F)	28/21	38/12	26/23
Age (mean ± SD)	35.2 ± 9.0	36.5 ± 8.9	31.7 ± 8.9

*BD, bipolar disorder patients; SC, schizophrenia patients; NC, Normal Control; M, Male; F, Female*.

Many preprocessing steps for the fMRI images were performed using Data Processing Assistant for Resting-State fMRI (DPARSF, http://pub.restfmri.net/) (Chao-Gan and Yu-Feng, 2010), Statistical Parametric Mapping (SPM12) (http://www.fil.ion.ucl.ac.uk/spm), and the Resting-State fMRI Data Analysis Toolkit (REST 1.8) packages (Song et al., 2011). These steps include slice time correction, brain skull removal, and motion correction followed by temporal pre-whitening, spatial smoothing, global drift removal, and band pass filtering. Specifically, the first 10 time points of each subject were removed; slice-timing correction and image realignment were carried out on the remaining time points. Because the brain size, shape, orientation, and gyral anatomy of each subject is different, the fMRI data of each subject was usually normalized into the Montreal Neurological Institute (MNI) space (resampled into 3 × 3 × 3 mm^3^ voxels) by using a unified segmentation on the T1 image. Then, the linear trends of the time courses were removed, and the effect of nuisance covariates was removed by signal regression using the global signal, the six motion parameters, the cerebrospinal fluid (CSF) and white matter (WM) signals. Temporal filtering (0.01 Hz < *f* < 0.08 Hz) was applied. Lastly, since we used only gray matter (GM) tissue to construct the functional connectivity network, the gray matter mask was used to mask the corresponding fMRI images to eliminate the possible effects from CSF and WM.

### Method

The core of our proposed method is listed below and will be described comprehensively in the following sections:

1. Labeling the DMN and generating the MST brain functional network.
2. Brain network similarity assessment.
3. Spectral clustering algorithm based on brain networks.

#### Labeling the DMN and Generating the MST Brain Network

Many studies have confirmed that the Default Mode Network (DMN) maintains a relatively stable state in the whole brain network, which is suitable for the study of the abnormality of the brain function network connections. In addition, a large number of studies have confirmed that AD patients have abnormal functional connections in the DMN (Mevel et al., 2011; Garcés et al., 2014). The connection abnormality is mainly reflected in the decrease of functional connections in the Posterior Cingulate Cortex (PCC) and Hippocampus (HIP), and the degree of reduction is positively correlated with the degree of episodic memory impairment. With the development of the disease, the impairment of DMN is aggravated gradually. Previous studies have confirmed that Bipolar disorder (Öngür et al., 2010), Schizophrenia (Mingoia et al., 2010; Tang et al., 2013) patients have abnormal functional connections in the DMN. Therefore, the connection abnormality of the DMN could provide an imaging marker for monitoring AD, BP, and SC.

(1) Labeling the DMN

In this paper, according to the Automated Anatomical Labeling (AAL) (Tzourio-Mazoyer et al., 2002) atlas in concordance with another study (Ciftçi, 2011), the DMN consisted of 32 locations and are shown in Table 3. These 32 locations were defined as the nodes of the brain network, and node time series were obtained by averaging the corresponding voxel time series in the anatomical areas. Then, with the Pearson correlation coefficients between pairs of nodes as connectivity weights, a functional full connected network was finally constructed for each subject.

**Table 3:** AAL structures forming the DMN.

**Region**	**Abbreviation**
Orbitofrontal cortex (superior)	ORBsup
Middle frontal gyrus	MFG
Orbitofrontal cortex (middle)	ORBmid
Rectus gyrus	REC
Anterior cingulate gyrus	ACG
Posterior cingulate gyrus	PCG
Precuneus	PCUN
Hippocampus	HIP
Parahippocampus gyrus	PHG
Inferior parietal lobule	IPL
Angular gyrus	ANG
Superior temporal gyrus	STG
Temporal pole (superior)	TPOsup
Middle temporal gyrus	MTG
Temporal pole (middle)	TPOmid
Inferior temporal gyrus	ITG

(2) Constructing the MST brain network

When building a brain network, the traditional approach is to convert a fully connected network into a binary network by setting a threshold. And there is no gold standard for the selection of thresholds. In addition, because different thresholds get different binary networks, this will affect the results of subsequent analysis to a certain extent. In order to avoid the threshold selection problem and preserve the structure of the brain network, we adopt the minimum spanning tree network correction scheme to construct the unbiased brain network. The MST method not only preserves the core framework of the network and ensures the neural interpretability of the network, but also eliminates the influence of the threshold. The MST network correction scheme has been widely applied to construct brain networks. For example, Guo et al. (2017) constructed minimum spanning tree high-order functional connectivity networks to identify AD from NC. van Dellen et al. (2018) constructed the MST structural brain networks of healthy adults, and concluded that MST was a feasible method to analyze structural brain networks. Cui et al. (2018) constructed the MST functional brain network for AD, MCI, and NC, analyzed the difference of topological structure among them, and classified them by using topological structural features.

In this paper, we constructed the MST brain network based on the full connected network by employing Kruskal's algorithm (Kruskal, 1956). The details of the algorithm used in this study are as follows: (1) order the weights of the full connected network in descending order; (2) link the nodes with maximal weight until all the nodes are linked in a loopless subgraph; (3) skip the link if the addition of this link leads to a loop.

In this study, the number of nodes in the topology of MST was 32 and the number of edges was 31.

#### Brain Network Similarity Measure

**Figure 2:**
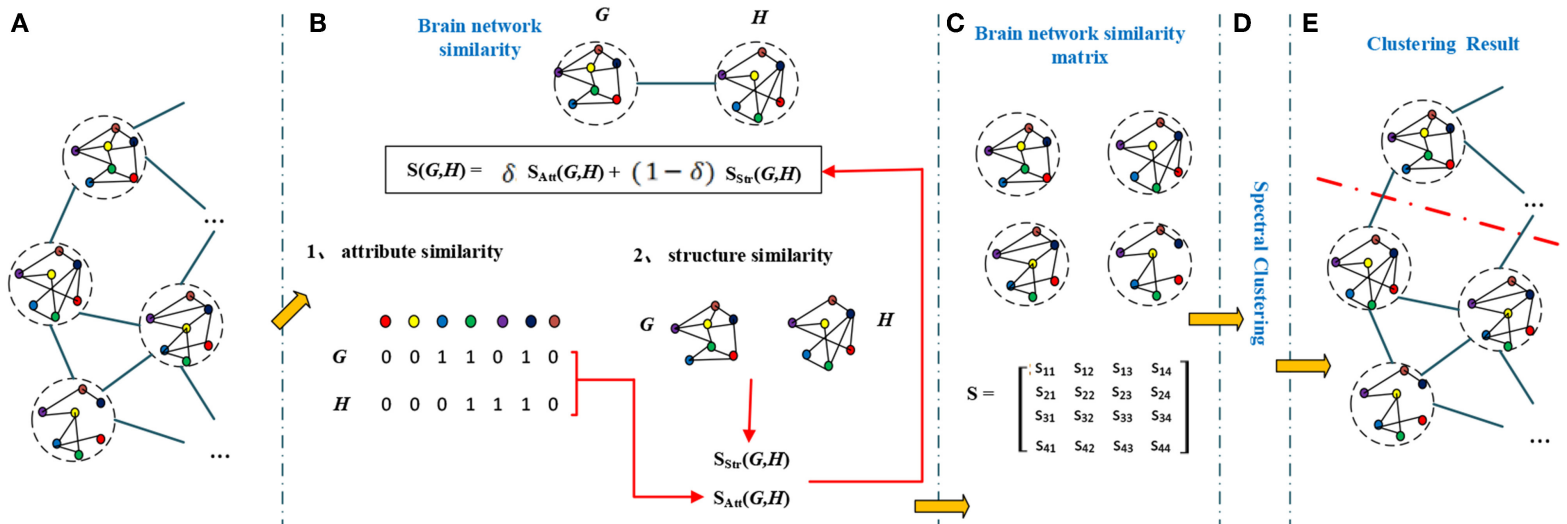
The brain network clustering framework. A graph **(A)** contains a number of inter-connected nodes, each node represents a brain network, different color represent different brain region. To calculate similarities between brain networks (G and H), we first find similarity between brain networks by taking node attributes (attribute similarity) and structures (structure similarity) into consideration **(B)**. The similarity matrix **(C)** is formed by the effective combination of attribute similarity and structural similarity. Similarity matrix and spectral clustering **(D)** results in final clustering results in **(E)**.

A brain network has not only attribute features but also topological features. So the similarity of brain networks was evaluated by their attribute similarity and structural similarity. The brain network clustering framework is shown in Figure 2.

1. Brain network attribute similarity

Betweenness is an important graph theoretical metrics in MSTs. In clinical application, betweenness centrality was used to compare brain networks of healthy subjects and patients with schizophrenia, depression and Alzheimer diseases (van den Heuvel et al., 2010; Yao et al., 2010; Becerril et al., 2011). Hence, the attribute similarity of brain networks is evaluated by measuring the similarity of betweenness. Betweenness of nodes is defined as the number of shortest paths through a node.

The betweenness *b*_*i*_ of the node *i* is defined as (Tewarie et al., 2015):

(1) $$\begin{array}{l} b_{i}=\frac{1}{\left(n-1\right)\left(n-2\right)}\underset{\begin{array}{c} h,j\in V\\ h\neq j,h\neq i \end{array}}{\sum }\frac{\rho_{hj}^{i}}{\rho_{hj}} \end{array}$$

where ρ_*hj*_ is the number of shortest paths between node *h* and *j*; $\rho_{hj}^{i}$ is the number of shortest paths between node *h* and *j* through the node *i*; *V* is the set of nodes; and *n* is the number of nodes.

The attribute similarity *s*_*att*_(*G, H*) is calculated using the cosine similarity method (Nguyen et al., 2011). The formula is as follows:

(2) $$\begin{array}{l} s_{att}\left(G,H\right)=\frac{\sum_{m=1}^{n}b_{m}\left(G\right)\times b_{m}\left(H\right)}{\sqrt{\sum_{m=1}^{n}{\left(b_{m}\left(G\right)\right)}^{2}}\times \sqrt{\sum_{m=1}^{n}{\left(b_{m}\left(H\right)\right)}^{2}}} \end{array}$$

where *b*_*m*_*(G)* is the betweenness of the *m*-th node in the brain network *G*, *b*_*m*_*(H)* is the betweenness of the *m*-th node in brain network *H*, and *n* is the number of nodes in the brain network.

2. Brain network structure similarity

A kernel can be seen as a measure of similarity between a pair of subjects. When a kernel is used for graph data, called a graph kernel, the data is mapped from the original graph space to the feature space and further measures the similarity between two graphs by comparing their topological structure (Shervashidze et al., 2011).

In this paper, sub-network kernels (Jie et al., 2018) were used to measure the topological structure similarity of brain networks. Compared with traditional graph kernels, sub-network kernels not only take into account the uniqueness of each node in brain networks, but also capture the multi-level topological properties of the brain network nodes.

The detailed process of sub-network kernels is summarized as follows:

(1) We construct a set of sub-networks on each node to reflect the connectivity of the brain network at multiple levels.

Specifically, *G* = (*V, E*) and *H* = (*V, E*′) represent a pair of brain networks, where *V* represents the node set for the networks. *E* and *E*′ represent the edge sets for *G* and *H*, respectively. Because the brain has the same brain area they share the same nodes.

To reflect the multi-level topological properties of brain networks, we first define two sets of sub-networks on each node *V*_*i*_ in the networks *G* and H,

(3) $$\begin{array}{l} G_{i}^{h}={\left\{G_{i}^{j}=(V_{\;i}^{j},E_{i\;}^{j})\right\}}_{j\;=\;1,2,\cdots ,\text{​}h}\\ H_{i}^{h}={\left\{H_{i}^{j}=({V^{\prime }}_{\;i}^{j},{E^{\prime }}_{\;i}^{j})\right\}}_{j=1,2,\cdots ,\text{​}h} \end{array}$$

where $V_{i}^{j}=\left\{v\in V|s(v,v_{i})\leq j\right\}$,$E_{i}^{j}=\left\{(u,v)\in E|u,v\in V_{i}^{j}\right\}$,$V_{i}^{\prime j}=\{v\in \;V|s(v,\;v_{i})\leq j\}$,$E_{i}^{\prime j}=\{(u,\;v)\in E\prime |u,\;v\in V_{i}^{\prime j}\}$ and *s*(·, *v*_*i*_) is the length of the shortest-path between node *V*_*i*_ and the other node. Here, *h* determines the maximum of *s*(·, *v*_*i*_) and also defines the number of sub-networks in the set $G_{i}^{h}$ and $H_{i}^{h}$.

According to Equation (3), for a brain network of *n* nodes, we can obtain *n* sets of sub-networks:

$$\begin{array}{l} G=\{G_{1}^{h}G_{2}^{h},\cdot \cdot \cdot ,\;G_{n}^{h}\}\\ H=\{H_{1}^{h}H_{2}^{h},\cdot \cdot \cdot ,\;H_{n}^{h}\} \end{array}$$

(2) We can calculate the kernel of brain networks *G* and *H* by calculating the similarity of all sub-network groups from the same node. The kernel of brain networks *G* and *H* is defined as:

(4) $$\begin{array}{l} k\left(G,H\right)=\frac{1}{n}\overset{n}{\underset{i=1}{\sum }}f\left(G_{i}^{h},H_{i}^{h}\right) \end{array}$$

with

(5) $$\begin{array}{l} f\left(G_{i}^{h},H_{i}^{h}\right)=\frac{1}{h}\overset{h}{\underset{j=1}{\sum }}g\left(G_{i}^{j},H_{i}^{j}\right) \end{array}$$

and

(6) $$\begin{array}{l} g\left(G_{i}^{j},H_{i}^{j}\right)=exp\left(-\frac{1}{2}\log\left(\frac{\left|{\text{A}}_{i}^{j}\right|}{\sqrt{\left|C^{G_{i}^{j}}||C^{H_{i}^{j}}\right|}}\right)\right) \end{array}$$

where |·| is the determinant, $C^{G_{i}^{j}}\in R^{d\times d}$ and $C^{H_{i}^{j}}\in R^{d\times d}$ are the corresponding covariance matrices which are defined on the sub-networks $G_{i}^{j}$ and $H_{i}^{j}$ by Equation (7) (Shrivastava and Li, 2014), respectively, *d* represents the number of power iterations, *n* denotes the number of nodes in the brain network, and ${\text{A}}_{i}^{j}$ is defined in Equation (8).

(7) $$\begin{array}{lll} C_{i,j}^{W} & = & cov\left(\frac{NW^{i}e}{||W^{i}e|{|}_{1}},\frac{NW^{j}e}{||W^{j}e|{|}_{1}}\right) \end{array}$$

(8) $$\begin{array}{lll} {\text{A}}_{i}^{j} & = & \frac{\left(C^{G_{i}^{j}}+C^{H_{i}^{j}}\right)}{2} \end{array}$$

where *CϵR*^*d* × *d*^ is a covariance matrix, *d* is the number of power iterations, *cov* denotes the covariance between two vectors, *W* denotes the adjacency matrix for the sub-network, *e* is the vector of all ones, and ‖·‖_1_ denotes the *l*_1_ norm of a vector. Here, the set of power iterations on a given vector *e*,{*e, We, W*^2^*e*, …, *W*^*d*^*e*}, is known as the “*d*-order Krylov subspace” which contains sufficient information to describe the adjacency matrix *W* for some appropriately chosen *d*.

Finally, the topological structural similarity between two brain networks *G* and *H* is equal to the kernel of the two brain networks *G* and *H*, and is defined as:

(9) $$\begin{array}{l} s_{str}\left(G,H\right)=k\left(G,H\right) \end{array}$$

3. Brain network similarity

Because the similarity of brain networks includes two parts (attribute similarity and structure similarity), it is necessary to combine them into one similarity. For this combination, we use a weight δ to control the degree of contribution of each part. In addition, since attribute similarity and structure similarity are two different types, normalization must be performed before combining them. The normalization is defined as:

(10) $$\begin{array}{l} s_{norm}\left(X,Y\right)=\frac{s\left(X,Y\right)}{\sqrt{s\left(X,X\right)s\left(Y,Y\right)}} \end{array}$$

The similarity *s*_*G,H*_ of two brain networks *G* and *H* is defined as follows:

(11) $$\begin{array}{l} s_{G,H}=\delta s_{att}\left(G,H\right)+\left(1-\delta \right)s_{str}\left(G,H\right) \end{array}$$

So similarity matrix for all brain networks **S** is defined as follows:

(12) $$\begin{array}{l} \text{S}=\left[\begin{array}{ccccc} s_{11} & \cdots & s_{1h} & \cdots & s_{1n}\\ \vdots & \ddots & \vdots & \ddots & \vdots \\ s_{g1} & \cdots & s_{gh} & \cdots & s_{gn}\\ \vdots & \ddots & \vdots & \ddots & \vdots \\ s_{n1} & \cdots & s_{nh} & \cdots & s_{nn} \end{array}\right] \end{array}$$

where *s*_*gh*_ represents the similarity between brain networks *G* and *H*, and *n* represents the number of brain networks. *s*_*gh*_ ranges between 0 (no similarity at all) to 1 (fully similar / same network).

#### Spectral Clustering Algorithm Based on Brain Networks

With the similarity matrix **S** obtained in the above section, we can formulate the clustering of brain networks as a spectral clustering (Ng et al., 2002; von Luxburg, 2007) problem, in which brain networks with a higher similarity tend to be grouped into the same cluster.

**Algorithm 1:** Spectral clustering algorithm based on brain networks

**Input**: A set of brain networks with each brain network an undirected graph; and cluster number *m*.
**Output**: *m* clusters *c*_1_*,c*_2_…*c*_*m*_
1. Initialize the similarity matrix **S** as an *n* × *n* zero matrix, *n* is the number of brain networks;
2. Form the brain network attribute similarity matrix *s*_*att*_ by Equation (2);
3. Form the brain network structure similarity matrix *s*_*str*_ by performing sub-network kernels;
4. Form the similarity matrix **S** defined by Equation (11) and Equation (12);
5. Define **D** to be the diagonal matrix whose (*i,i*)-element is the sum of **S**'s *i*-th row, and construct the matrix **L** = **D**^−1/2^**S** **D**^−1/2^;
6. Find *x*_1_, *x*_2_⋯ , *x*_*m*_, the *m* largest eigenvectors of **L**, and form the matrix $\text{X}=\left[x_{1},x_{2}\cdots \;,x_{m}\right]\in R^{n\times m}\;$by stacking the eigenvectors in columns;
7. Form the matrix **Y** from **X** by renormalizing each of **X**'s rows to have unit length;
8. Treating each row of **Y** as a point in *R*^*m*^, cluster them into *m* clusters via the K-means algorithm;
9. Finally, assign the brain network to cluster *c*_*i*_ if and only if row *i* of the matrix **Y** was assigned to cluster *c*_*i*_.

#### Methodology

In all algorithms, we set the number of clusters to 2 for classifying the patients and the healthy controls. In addition, there were certain parameters that needed to be set in the proposed algorithm: (1) We apply grid search to find the optimal value for δ. We do grid search for δ in {0.1, 0.2, … 0.9}. (2) In the sub-network kernels, the parameters *h* and *d* are set to 3 and 3 for AD, the parameters *h* and *d* are set to 3 and 1 for SC and BP, respectively.

To evaluate the consistency between the clustering labels and clinical diagnostic labels of the subjects, we defined clustering consistency as similar to clustering accuracy (Wang et al., 2010), which can be used to discover one to one relationships between clusters and clinical classes and can measure the extent to which each cluster contains data points from the corresponding class.

Clustering consistency sums up the entire matching degree between all pair class clusters. Clustering consistency can be computed as

(13) $$\text{consistency=}\frac{\text{1}}{\text{n}}\text{max}(\sum_{{\text{C}}_{\text{m}},{\text{L}}_{\text{p}}}\text{T}({\text{C}}_{\text{m}},{\text{L}}_{\text{p}}))$$

where C_*m*_ denotes the *m*-th (*m*[{1, 2}) cluster in the final results, and L_*p*_ is the diagnostic *p*-th (*p*[{1, 2}) group (patient group and control group). T(C_*m*_, L_*p*_) is the number of samples that belong to group *p* and are assigned to cluster *m*. *n* represents the number of brain networks. Consistency is the maximum sum of T(C_*m*_, L_*p*_) for all pairs of clusters and groups, and these pairs have no overlaps.

## Results

The minimum spanning tree brain network of the default mode network was constructed using Kruskal's algorithm. Then, According to a certain proportion, the attribute similarity matrix and the structure similarity matrix were combined to form a similarity matrix which is used for clustering. Finally the clustering of the brain network was completed by spectral clustering.

### Clustering Performance

In order to evaluate the clustering performance of our proposed method, we compared our method with methods that use a different similarity measure for the same dataset, including:

1. Spectral clustering algorithm based on node attributes: It is an existing similarity measure of brain networks only considering node attribute similarity. We first constructed the similarity matrix only based on node betweenness and then used the similarity matrix as the input for normalized spectral clustering.
2. Spectral clustering algorithm based on kernel method (Jie et al., 2018): It is an existing similarity measure of brain networks only considering structure distance between two brain networks. We first constructed the similarity matrix for graphs based on the kernel method and then used the similarity matrix as the input for normalized spectral clustering.
3. Spectral clustering algorithm based on SimiNet (Mheich et al., 2018): SimiNet takes into account the physical locations of nodes and the weight difference of edge when computing similarity between two brain graphs. We first constructed the similarity matrix for graphs based on SimiNet and then used the similarity matrix as the input for normalized spectral clustering.

In all experiments, we evaluate the performance of methods by measuring clustering consistency. Clustering consistency is used to find one-to-one relationships between clusters and clinical classes of the subject, and to measure the extent to which each cluster contains data points from the same class. Table 4 shows the clustering performances of the different methods with the same dataset. The results showed that our proposed method achieved the best clustering performance, with a consistency of 60.6% for AD, with a consistency of 100% for SC, with a consistency of 100% for BP.

**Table 4:** Clustering performance of different similarity measure.

**Dataset**	**Similarity** **measurement**	**Consistency**		
		**Patient (%)**	**Control (%)**	**Total (%)**
AD	Node attribute	64.6	54.1	58.7
	Kernel method	60.4	39.3	48.6
	Siminet	58.3	59	58.7
	Our method	**62.5**	**59**	**60.6**
SC	Node attribute	90.0	77.6	83.8
	Kernel method	98.0	100	98.9
	Siminet	58.0	55.1	56.6
	Our method	**100**	**100**	**100**
BP	Node attribute	53.1	46.9	50
	Kernel method	100	100	100
	Siminet	63.3	51.0	57.1
	Our method	**100**	**100**	**100**

*The bolded values represent the clustering performance of our method*.

## Discussion

### Performance Evaluations

The clustering performance of four different similarity measurement methods is listed in Table 4. When the consistency is 100%, the clustering label of all subjects is consistent with the clinical diagnostic label, and the clustering accuracy is 100%. When the consistency is 0%, the clustering label of all subjects is inconsistent with the clinical diagnosis label, and the clustering accuracy is 0%.

As shown in Table 4, among the four similarity measure methods, our method performed the best on the three datasets in terms of consistency. The node attribute and the kernel methods achieved a slightly better result.

The node attribute method directly used the node attributes of a brain network for calculating the similarity between each pair of brain networks, which was utilized for the final brain network clustering. The result shows that the similarity of the brain network cannot be more accurately determined from the node attributes (betweenness) alone. The result shows that the brain disorders are associated with alterations in the hubs. Many studies have also demonstrated that the hubs of the human brain are generally implicated with brain disorders (He et al., 2008; Lynall et al., 2010), such as AD and SC.

The kernel method computed the similarity matrix by performing sub-network kernels on the brain network. The result means that when the attributes of the network change, this will affect the global connection structure of the network. Therefore, the description of the global structure has a great effect on the clustering.

SimiNet measures the similarity between the two graphs according to the node and edge attributes under the spatial constraints related to the physical position of the nodes. The key feature of this algorithm is that it takes into account the physical locations of the network nodes. However, in the not-weight brain network constructed with rs-fMRI (resting state fMRI) data, the position of the nodes is the same, so the advantages of the algorithm are not fully reflected. As shown in Table 4, we can see that this method achieved a slightly better result.

Different to the above methods, our method combines both the attribute similarity and structure similarity, where the attribute similarity captures the topological characteristics of brain networks and the structure similarity captures the structure distance. In addition, sub-network kernels were used to measure the structural similarity of brain networks. It not only takes into account the uniqueness of each node, but also captures the multi-level topological properties of nodes in the networks, which are essential for defining the similarity measure. These results indicated that the attribute features and the interior-node structure were important for graph clustering. So, the similarity measurement method based on the combination of attributes and structure can accurately describe the similarity of the brain network, thus improving the clustering performance.

In addition, the results show that when the method is applied to different data sets, the clustering performance is also different, which indicates that the clustering performance is affected by the data to a certain extent. This is because we choose DMN as regions of interesting to construct brain network in this study. The damage degree of DMN is different in different brain diseases, which affects the performance of clustering.

### Effect of Parameters *δ, d*, and *h*

**Figure 3:**
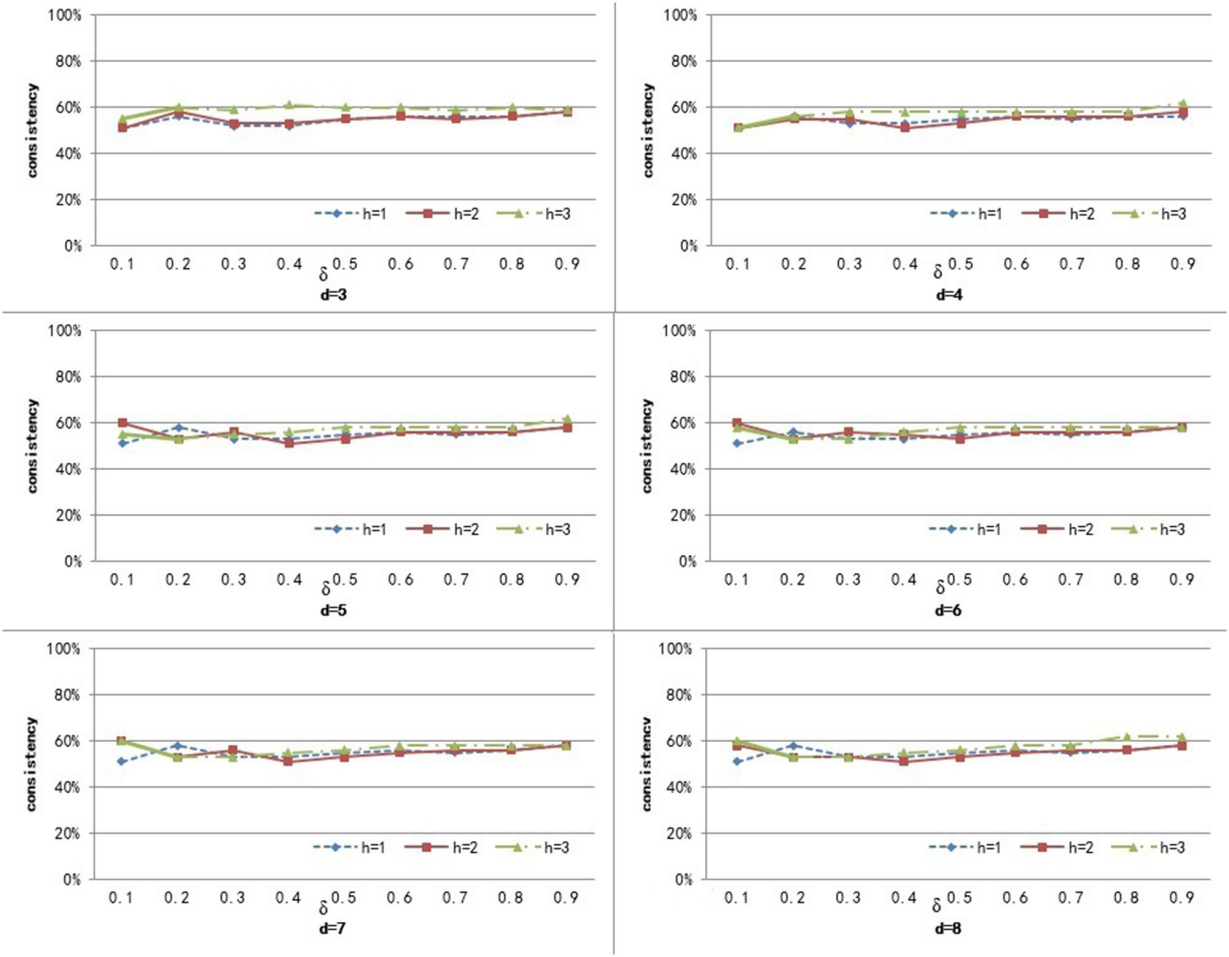
The cluster result of AD with respect to different values of parameter δ, *h*, and *d*.

**Figure 4:**
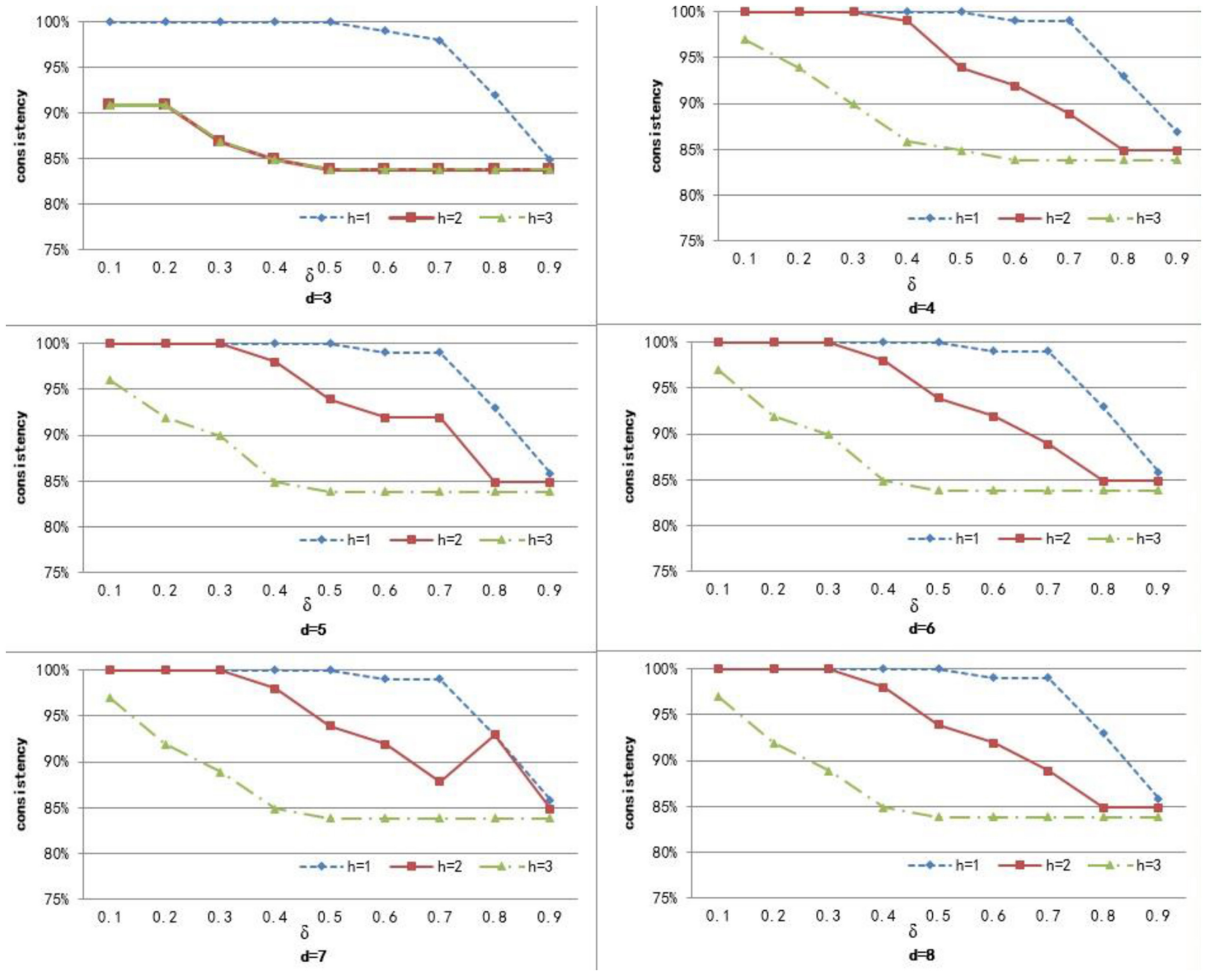
The cluster result of SC with respect to different values of parameter δ, *h*, and *d*.

**Figure 5:**
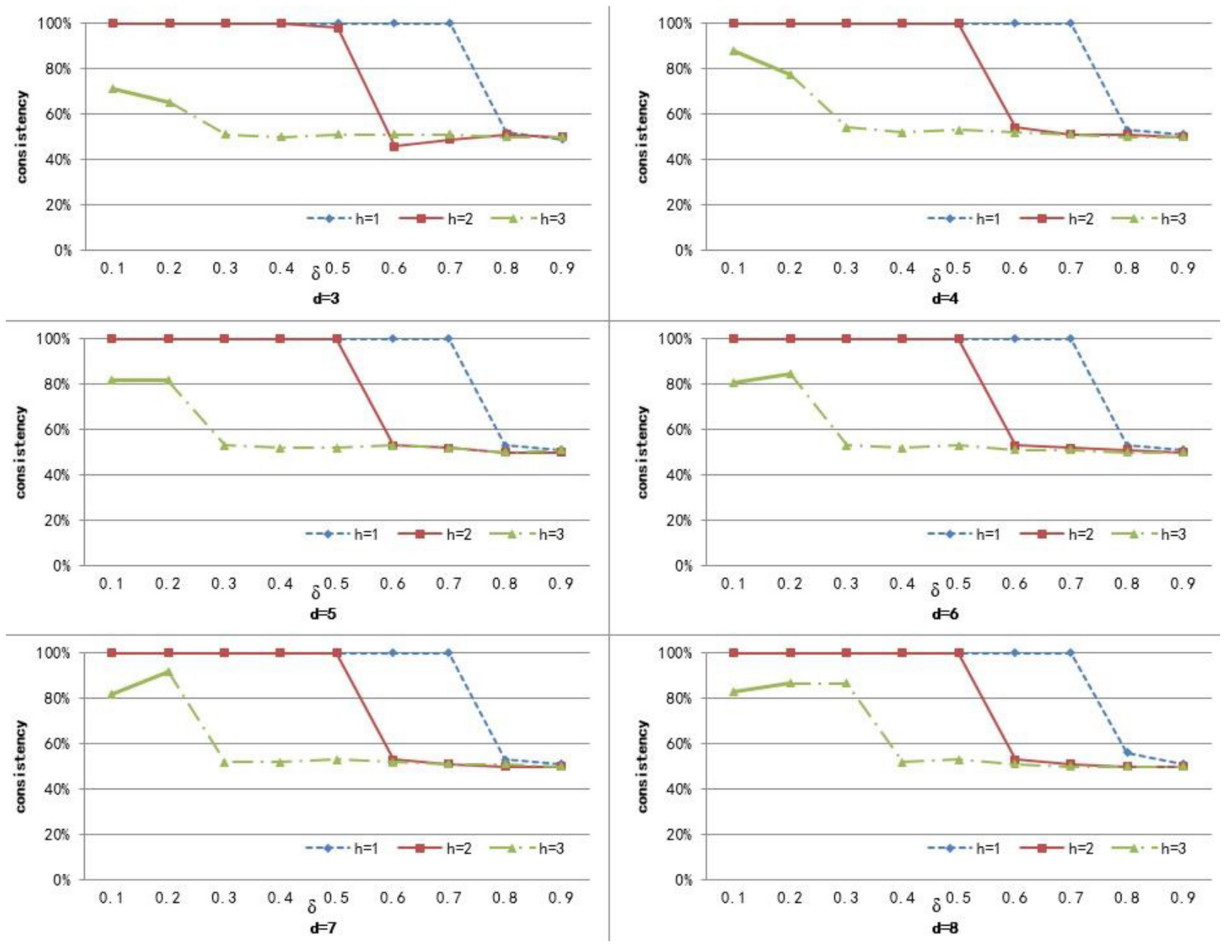
The cluster result of BP with respect to different values of parameter δ, *h*, and *d*.

To compute the similarity of two graphs, the parameters δ, *d*, and *h* need to be set. *d* controls the number of power iterations, and *h* is the size of a sub-network set. The weight δ is used to control the degree of contribution of attribute similarity and structure similarity. In this section, we explore the effect of parameters δ, *d*, and *h* on clustering performance. To analyze the effect of these parameters on our method, we set different values for *d* ∈ {3, 4, 5, 6, 7, 8} and *h* ∈ {1, 2, 3}, and δ was set from 0.1 to 0.9 with a step of 0.1. Figures 3–5 shows the clustering results of AD, SC, and BP with respect to different values of these parameters.

From Figure 3 we can see that the consistency for AD is between 51 and 60.6%. The best clustering performance was obtained when *h* = 3 and δ = 0.9, with the consistency of 60.6%. From Figure 4 we can see that the consistency is between 84 and 100% for SC. The best clustering performance was obtained when *h* = 1 and ∈ [0.1, 0.7], with the consistency of 100%. From Figure 5 we can see that the consistency is between 50 and 100% for BP. The best clustering performance was obtained when *h* = 1 and δ ∈ [0.1, 0.7], with the consistency of 100%.

Figures 3–5 shows that, with a fixed *h*, the curves varied with the value of *d* are very smooth, which shows that our method is very robust to the parameter *d*. Moreover, we can observe that, given a fixed *d*, the clustering performance is largely affected by different values of *h*. When *h* = 1, our method obtains the best clustering performance for SC and BP. When *h* = 3, our method obtains the best clustering performance for AD. These results imply that the selection of *h* is critical for our proposed method. This is reasonable since the number *h* controls the size of a sub-network set for each node in a brain network, and thus affects the similarity measurement of brain networks. In additional, these results imply that the selection of δ is critical for our proposed method. It is because that δ controls the degree of contribution of attribute similarity and structure similarity.

The experimental results showed that the topological structure and the attribute features of brain networks play important roles in clustering brain networks. The setting of parameters is related to the experimental data.

### Limitation

Although the proposed method is effective, when this method is applied to different datasets, the clustering performance is different, which indicates that the clustering performance is affected by the data to a certain extent. In addition, the proposed method does not take into account a priori knowledge of the subject, such as Mini-Mental State Examination and Clinical Dementia Rating. A large number of studies have shown that making full use of a priori knowledge in the process of searching for clusters can significantly improve the performance of the clustering algorithm (Jiao et al., 2012). Therefore, it will be meaningful to combine this knowledge with spectral clustering.

## Conclusion

In this paper, we proposed a framework for spectral clustering based on attribute feature similarity and topological structure similarity. Specifically, we use cosine similarity to measure the attribute similarity between brain networks. Then, we use sub-network kernels to calculate the structure similarity between brain networks. Finally, according to an optimal parameter δ, the similarity matrix was obtained by integrating the structure similarity and attribute similarity, and spectral clustering is carried out. Hence, this new similarity matrix considers both the global and local similarity of brain networks. In experiments with the AD, BP, and SC dataset, we demonstrated that our proposed method can significantly improve clustering performance in terms of consistency. In our future work, we will explore the combination of a priori knowledge and spectral clustering and carry out further research in this area.

## Data Availablity Statement

The data used in this study was from three datasets. (1) Alzheimer's Disease Neuroimaging Initiative (ADNI) database, accessible at: http://adni.loni.usc.edu/. (2) Obtained from the public database openfMRI, accessible at: https://www.openfmri.org/).

## Ethics Statement

The grantee organization of ADNI project is the Northern California Institute for Research and Education (NCIRE), and the study is coordinated by the Alzheimer's Therapeutic Research Institute (ATRI) at the University of Southern California. ADNI data are disseminated by the Laboratory of Neuro Imaging at the University of Southern California. This research was also supported by NIH grants P30 AG010129 and K01 AG030514. All ADNI subjects together with their legal representatives should have written informed consent before collecting clinical, genetic and imaging data.

The OpenfMRI project is managed by the Poldrack Lab and Center for Reproducible Neuroscience at Stanford University, with computing resources provided by the Texas Advanced Computing Center and Amazon.com. It is funded by grants from the National Science Foundation, National Institute for Drug Abuse, and Laura and John Arnold Foundation.

## Author Contributions

XC proposed a framework of spectral clustering based on brain network. JXiao, YN, and DL processed data and made experiment. JXian, BW, HG, and JC gave the proof of results.

### Conflict of Interest

This manuscript has not been published or presented elsewhere in part or in entirety, and is not under consideration by any another journal. Meanwhile, all the authors have read through the manuscript, approved it for publication, and declared no conflict of interest.
